# Supplementary material for: The elemental mechanism of transcriptional pausing
Source: eLife. 2019 Jan 8;8:e40981. doi: 10.7554/eLife.40981 (PMC6336406; doi:10.7554/eLife.40981)
Supplement: Supplementary file 1. [file elife-40981-supp1.pdf]

Supplementary file 1. Oligonucleotides and plasmids used in this study.

Oligonucleotides	Source	Lab Stock #
ePEC non-template DNA (consensus)<br>GGTCAGTACGTCCGGCATAGTTGCGCCCGTAAATTCAGATCTTCC AGTGG	IDT	#9563
ePEC non-template DNA (bubble scaffold)<br>GGTCAGTACGTCCGGTCGATCTTCGCCCGTAAATTCAGATCTTCC AGTGG	IDT	#8333
ePEC template DNA (consensus)<br>CCACTGGAAGATCTGAATTTACGGGCGCAACTATGCCGGACGTA CTGACC	IDT	#8334
ePEC RNA -11G (consensus)<br>UUUUUUGGCAUAGUU	IDT	#8342
ePEC RNA -11U<br>UUUUUUUGCAUAGUU	IDT	#10797
ePEC non-template DNA (Bochkareva *et al*., 2012)<br>ACTTACAGTAATCCGAAGTACACACGGCGAATAGCCA	IDT	#8195
ePEC template DNA (Bochkareva *et al*., 2012)<br>TGGCTATTCGCCGTGTGTACTTCGGATTACTGTAAGT	IDT	#8196
ePEC non-template long DNA (Bochkareva *et al*., 2012)<br>ACTTACAGTAATCCGAAGTACACACGGCGAATAGCCATTCCAGTG G	IDT	#8289
ePEC template long DNA (Bochkareva *et al*., 2012)<br>CCACTGGAATGGCTATTCGCCGTGTGTACTTCGGATTACTGTAAG T	IDT	#8290
ePEC RNA (Bochkareva *et al*., 2012)<br>AAUAAUCCGAAGU	IDT	#7974
ePEC tandem scaffold non-template DNA<br>GGTACCGGCATAGTTGCGCCCGGCAAAGATGCGCCCGTAAATTC AGATCTTCCAGTGG	IDT	#12623
ePEC tandem scaffold template DNA<br>CCACTGGAAGATCTGAATTTACGGGCGCATCTTTGCCGGGCGCA ACTATGCCGGTACC	IDT	#12624
EC non-template DNA for DS-6MI control<br>GGTGATCTAAGGTTCCAGAGGAGCGXTCATGTCAGTAGAGTAGC GAGTGG X=6-MI	Fidelity Systems	#11159
EC template DNA for DS-6MI control<br>CCACTCGCTACTCTACTGACATGACCGCTCCTCTGGAACCTTAGA TCACC	IDT	#11242
EC RNA for DS-6MI control<br>CUCACAACCAGAGGAG	IDT	#8106
ePEC non-template DNA for DS-6MI pause<br>GGTCAGTACGTCCGGCATAGTTGCGXTCGTAAATTCAGATCTTCC AGTGG	Fidelity Systems	#11156

ePEC template DNA for DS-6MI pause<br>CCACTGGAAGATCTGAATTTACGACCGCAACTATGCCGGACGTAC<br>TGACC	IDT	#11241
ePEC RNA for DS-6MI pause<br>UUUUUUGGCAUAGUUG	IDT	#8401
ePEC non-template DNA for US-6MI control<br>ACTTGTAGCGATCTAAGGTTCCAGAGGAGCGGTCATGTCAGTAGA<br>GTAGC	IDT	#8102
ePEC template DNA for US-6MI control<br>GCTACTCTACTGACATGACCGCTCCTCTXGAACCTTAGATCGCTA<br>CAAGT	Fidelity Systems	#8110
ePEC non-template DNA for US-6MI pause<br>GGTCAGTACGTCCGCCATAGTTGCGCCCGTAAATTCAGATCTTCC<br>AGTGG	IDT	#8399
ePEC template DNA for US-6MI pause<br>CCACTGGAAGATCTGAATTTACGGGCGCAACTATXGCGGACGTA<br>CTGACC	Fidelity Systems	#8385
ePEC RNA for US-6MI pause<br>UUUUUUGCCAUAGUUG	IDT	#8402
ePEC template DNA (usFJ variant)<br>CCACTGGAAGATCTGAATTTACGGGCGCAACTATGGCGGACGTA<br>CTGACC	IDT	#9762
ePEC RNA (usFJ variant)<br>UUUUUUGCCAUAGUU	IDT	#8763
ePEC template DNA (Hyb variant)<br>CCACTGGAAGATCTGAATTTACGGGCGCGAGTATGCCGGACGTA<br>CTGACC	IDT	#11639
ePEC RNA (Hyb variant)<br>UUUUUUGGCAUACUC	IDT	#11640
ePEC non-template DNA (dsFJ variant)<br>GGTCAGTACGTCCGGCATAGTTGTGCCCGTAAATTCAGATCTTCC<br>AGTGG	IDT	#10806
ePEC non-template DNA (DSDNA variant)<br>GGTCAGTACGTCCGGCATAGTTGCGGTCGTAAATTCAGATCTTCC<br>AGTGG	IDT	#11178
ePEC template DNA (DSDNA variant)<br>CCACTGGAAGATCTGAATTTACGACCGCAACTATGCCGGACGTAC<br>TGACC	IDT	#11241
ePEC template DNA (usFJ+Hyb variant)<br>CCACTGGAAGATCTGAATTTACGGGCGCGAGTATGGCGGACGTA<br>CTGACC	IDT	#11951
ePEC RNA (usFJ+Hyb variant)<br>UUUUUUGCCAUACUC	IDT	#11952
ePEC non-template DNA (usFJ+dsFJ variant)<br>GGTCAGTACGTCCGCCATAGTTGTGCCCGTAAATTCAGATCTTCC<br>AGTGG	IDT	#11533

ePEC non-template DNA (usFJ+DSDNA variant)<br>GGTCAGTACGTCCGCCATAGTTGCGGTCGTAAATTCAGATCTTCC<br>AGTGG	IDT	#11534
ePEC template DNA (usFJ+DSDNA variant)<br>CCACTGGAAGATCTGAATTTACGACCGCAACTATGGCGGACGTAC<br>TGACC	IDT	#11535
ePEC non-template DNA (Hyb+DSDNA variant)<br>GGTCAGTACGTCCGGCATAGTTGCGGTCGTAAATTCAGATCTTCC<br>AGTGG	IDT	#11178
ePEC non-template DNA (dsFJ+DSDNA variant)<br>GGTCAGTACGTCCGGCATAGTTGTGGTCGTAAATTCAGATCTTCC<br>AGTGG	IDT	#11537
ePEC non-template DNA (Double usFJ variant)<br>GGTCAGTACGTCCGTCATAGTTGCGCCCGTAAATTCAGATCTTCC<br>AGTGG	IDT	#10800
ePEC template DNA (Double usFJ variant)<br>CCACTGGAAGATCTGAATTTACGGGCGCAACTATGACGGACGTA<br>CTGACC	IDT	#10801
ePEC RNA (Double usFJ variant)<br>UUUUUUUUCAUAGUU	IDT	#10798
*his*PEC1 non-template DNA<br>GCGTCCTATCGATCTTCGGAAGAGATTCAGAG	IDT	#10924
*his*PEC1 template DNA<br>CTCTGAATCTCTTCCAGCACACATCAGGACGC	IDT	#10919
*his*PEC1 RNA (pause -2 RNA)<br>UCAUCCGGCGAUGUGUG	IDT	#6593
*his*PEC1 exit-channel duplex RNA<br>CCGGAUGA	IDT	#6594
Control EC2, *his*PEC2 non-template DNA<br>GGTCAGTACGTCCTGTCGATCTTCGGAAGAGAATTCAGATCTTCC<br>AGTGG	IDT	#8847
Control EC2 template DNA<br>CCACTGGAAGATCTGAATTCTCTTCCCCTCTAGCTCAGGACGTAC<br>TGACC	IDT	#8848
Control EC2 RNA<br>UUUUUUUGAGCUAGAGG	IDT	#8855
*his*PEC2 template DNA<br>CCACTGGAAGATCTGAATTCTCTTCCAGCACACATCAGGACGTAC<br>TGACC	IDT	#8851
*his*PEC2 RNA (pause RNA)<br>CCUGACUAGUCUUUCAGGCGAUGUGUGCU	IDT	#4865

**Plasmids**	Source	Lab Stock #
pRM756<br>RNAP($\alpha_2\beta\beta'\omega$) overexpression plasmid. His10-ppx tag at *rpoC*-C-terminus	Windgassen *et al.*, 2014	#2956
pRM843<br>RNAP($\alpha_2\beta\beta'\omega$) overexpression plasmid. HMK-Strep tag at *rpoC*-C-terminus, *rpoB*-His10-ppx.	Hein *et al.*, 2014	#5143
pRM1151<br>Derivative of pRM843. RNAP($\alpha_2\beta\beta'\omega$) overexpression plasmid containing *rpoB* substitution R542A.	This work	#5451
pRM 1147<br>Derivative of pRM756. RNAP($\alpha_2\beta\beta'\omega$) overexpression plasmid containing *rpoC* substitution K334A	This work	#5447
pDJ115<br>RNAP($\alpha_2\beta\beta'\omega$) overexpression plasmid containing β' substitutions I937C- P1139C with His_{10} and HMK tags at β´ C-terminus	Nayak *et al.*, 2013	#4954
pDJ116<br>RNAP($\alpha_2\beta\beta'\omega$) overexpression plasmid containing β' substitutions I937C- T1135C with His_{10} and HMK tags at β´ C-terminus	Nayak *et al.*, 2013	#4955
pDJ124<br>RNAP($\alpha_2\beta\beta'\omega$) overexpression plasmid containing β' substitution I937C- Q736C with His_{10} and HMK tags on the β´ C-terminus	Nayak *et al.*, 2013	#4963
pDJ129<br>RNAP($\alpha_2\beta\beta'\omega$) overexpression plasmid containing β' substitution I937C- G687C with His_{10} and HMK tags on the β´ C-terminus	Nayak *et al.*, 2013	#4968
pRM950<br>RNAP($\alpha_2\beta\beta'\omega$ overexpression plasmid containing substitutions β′258iC, β1045iC, and β 843C. HMK-Strep tag at β′ C-terminus, His_{10}-precission protease tag at β N-terminus.	Kang *et al.*, 2018a	#5250
pRM918<br>RNAP(α2ββ´ω) overexpression plasmid containing substitutions β′258iC. β1044C. HMK-Strep tag at β′ C-terminus, His_{10}-precission protease tag at β N terminus	Hein *et al.*, 2014	#5218

X , 6-Methylisoxanthopterin.
